# The risk of arterial thrombosis in carriers of natural coagulation inhibitors: a prospective family cohort study

**DOI:** 10.1007/s11739-021-02656-5

**Published:** 2021-02-22

**Authors:** Daniela Tormene, Franco Noventa, Elena Campello, Sabrina Gavasso, Michelangelo Marobin, Giacomo Turatti, Paolo Prandoni, Paolo Simioni

**Affiliations:** 1 Chair of Internal Medicine, Thrombotic and Haemorrhagic Diseases Unit, Department of Medicine, University of Padua Medical School, Padua, Italy; 2 Arianna Foundation on Anticoagulation, Bologna, Italy

**Keywords:** Arterial thrombosis, Inherited thrombophilia, Deficiency of natural coagulation inhibitors, Cardiovascular disease, Ischemic stroke

## Abstract

**Background:**

Whether the carriership of inherited antithrombin (AT), protein C (PC), and protein S (PS) deficiency increases the risk of arterial thromboembolic events (ATE) is controversial. This information has the potential to inform the management of family members of probands with inherited deficiency of natural anticoagulants.

**Patients/methods:**

We conducted a large prospective family cohort study in 640 subjects (of whom 341 carriers and 299 non-carriers) belonging to 86 families with inherited deficiency of AT, PC, or PS.

**Results:**

A total of 4240 and 3810 patient-years were available for carriers and non-carriers, respectively. Risk factors for atherosclerosis were similarly distributed in the two groups. Of the 26 ATE that were recorded, 19 occurred in carriers (5.6%), as compared to 7 in non-carriers (2.3%) [*p* = 0.07]. After adjusting for confounders, the hazard ratio (HR) for ATE was 4.9 (95% CI 1.5–16.3) in carriers as compared to non-carriers.

**Conclusions:**

Among family members of probands with an inherited deficiency of natural anticoagulants, carriers exhibit a risk of ATE that is almost five times higher than in non-carriers.

## Introduction

While inherited defects of antithrombin (AT), protein C (PC), and protein S (PS) are well established risk factors for venous thromboembolism (VTE) [1–3], whether they play a role in the development of ATE as well is still a matter of debate [4, 5]. As, in recent years, a number of factors have been reported to account, although not consistently, for the development of both venous and arterial thrombotic complications[6], we decided to assess whether the inherited deficiency of natural anticoagulants exposes carriers to the risk of arterial thrombotic events [7–12]. To this purpose, in a large prospective study, we assessed the risk of ATE in family members of probands with defects of AT, PC, and PS. The rate of events occurring in carriers was compared with that occurring in non-carriers. In the same cohort, we have previously reported the data on the risk of venous thromboembolism (VTE) [13]. This article is not currently available.

## Materials and methods

### Study population

Among all consecutive patients who referred to the Thrombosis Unit of the University Hospital of Padua between May 1993 and June 2009 with objectively confirmed symptomatic VTE, those who were identified as being carriers of inherited defects of AT, PC, or PS were labelled as the study probands. Their family members were identified by pedigree analysis. The first- and second-degree relatives were eligible for the study, provided that they were 15 years or older.

Prior to collecting blood sample for the identification of the deficiency status, they were interviewed with the use of a standardized questionnaire. Close attention was paid to the occurrence of previous episodes of venous and arterial thromboembolism and to the presence of cardiovascular risk factors.

The presence of a family history for cardiovascular risk factors was equally distributed between carriers and non-carriers of anticoagulant deficiency.

Hypertension was defined as a systolic blood pressure of ≥ 140 mm Hg or ≥ 160 mm Hg in patients ≥ 60 years of age, a diastolic blood pressure of ≥ 90 mm Hg, or the use of antihypertensive drugs. Hyperlipidemia was defined as a total cholesterol level > 250 mg/dL and triglycerides > 220 mg/dL or by the use of lipid lowering drugs [14]. Diabetes was diagnosed according to the guidelines provided by the Expert Committee on Diagnosis and Classification of this disease [15] or by the use of hypoglycaemic diet or glycaemia lowering drugs. Cigarette smoking and BMI were considered.

Patients with previous ATE or VTE were excluded, as were those with antiphospholipid syndrome, active malignancy, or illicit drug use.

Investigators blinded to the status of the study participants (carriers vs non-carriers), visited or interviewed by telephone all consenting subjects every 6 months, or earlier if symptoms or signs consistent with the development of ATE had developed. If this was the case, they were instructed to undergo objective diagnostic tests as appropriate. The follow-up ended in June 2014.

The study protocol was performed in agreement with the Declaration of Helsinki, and informed consent was obtained from all participant. The study protocol was approved by the medical Ethical Board of the University of Padua.

### Laboratory investigations

#### Routine tests

Nine milliliters of venous blood was collected from patients and controls, using 21-gauge needles without any venostasis, directly into syringes pre-filled with 1 mL of sodium citrate 109 mol/L. The first few milliliters were discarded to avoid the contact phase activation. Platelet poor plasma (PPP) was prepared within 1 h of blood collection by double centrifugation (2 × 15 min at 2500*g*) at room temperature. Coagulation tests, including activated partial thromboplastin time (PTT n.v., 24.4–36.5 s) and prothrombin time (PT n.v. 70–100%), were measured using Dade^®^ Actin^®^ Activated Cephaloplastin and Thromborel^®^ S reagents, respectively, on the BCT-Analyser (Siemens, Marburg, Germany). Aliquots (1.5 ml) were immediately frozen and then stored at − 80 °C until use. Samples were thawed by incubation for 5 min in a water bath at 37 °C immediately before assay. Samples were analysed only after a single freeze–thaw cycle, and repeated freeze–thaw cycles were avoided. Patient and control samples were all processed in the same way by the same experienced operators.

### Coagulation tests for thrombophilia

Antithrombin activity (n.v. 80–120%) was detected using a thrombin-based chromogenic substrate assay from Roche Diagnostics GmbH (Mannheim, Germany). Protein C chromogenic (n.v. 70–130%) and coagulometric (n.v. 80–120%) activities were measured using two commercial kits: Berichrom^®^ Protein C and Protein C Reagent (Siemens Healthcare Diagnostics, Marburg, Germany), respectively. The above tests were performed on a BCS XP coagulation analyzer (Siemens Healthcare Diagnostics, Germany). Protein S coagulometric activity (n.v. 70–130%) was assessed using the ProS kit (Instrumentation Laboratory, Milan, Italy) on an ACL TOP 300 CTS coagulation analyzer (Instrumentation Laboratory, Italy). AT antigen (n.v. 80–120%) was determined by a home-made enzyme-linked immunosorbent assay (ELISA), as previously described [16]. Protein C antigen (n.v. 80–120%) was determined by a home-made sandwich ELISA using a sheep anti-human protein C antibody (Affinity Biologicals, Ancaster, Ontario, Canada) as the capture antibody and a horseradish peroxidase (HRP)-conjugated sheep anti-human protein C antibody (Affinity Biologicals, England) as the detection antibody. Total (n.v. 80–120%) and free protein S antigen (n.v. 80–120%) were measured with a home-made ELISA as previously described.[17]

### Laboratory criteria for the diagnosis of inherited clotting inhibitor defects

The criteria used for the classification of the different types of AT, PC, and PS defects were those previously reported [2, 18]. Subjects who presented with the same defect in two consecutive laboratory determinations performed at an interval of at least 1 month were considered to be potentially carriers of an inherited defect. To confirm the inheritance, the same defect has to be present in at least one first-degree family member. Where possible, sequencing of AT, PC, and PS genes was performed to identify the lesions responsible for the defects and confirm the hereditary pattern (40% of patients). In women under oestrogen treatment or during pregnancy, blood samples were collected at least 3 months after withdrawal of hormonal treatment or delivery, respectively. In this study, we included only quantitative defects. Type I defects (concomitant reduction of antigen and activity to about 50%) were considered for Antithrombin and Protein C deficiency and type I (concomitant reduction of total and free protein S antigen and protein S activity to about 40–50%) and type III defects (normal total protein S antigen reduced free protein S antigen and protein S activity to about 40–50%) for Protein S deficiency.

### Outcomes

The primary outcome was the occurrence of symptomatic and objectively documented ATE. Diagnosis of peripheral artery thrombosis was accepted in the presence of acute signs and symptoms of ischemia and a positive arteriography. Myocardial infarction was confirmed by typical ECG features, elevated levels of cardiac enzymes, or coronary angiography. Ischemic stroke was documented by computed tomography scanning or magnetic resonance imaging. A transient ischemic attack required neurological signs and symptoms resolved within 24 h. All the diagnoses were adjudicated by fellow specialists in vascular, cardiological, and neurological fields. Hospital discharge letter and the related clinical documentation were reviewed, and the outcome adjudication was blind to the carriers and non-carriers status.

### Statistical analysis

The absolute risk of first arterial thrombosis was calculated in relatives, comparing those who did or did not have AT, PC, or PS defects. The relative risk (RR) for the development of ATE was calculated by dividing the incidence rate in carriers by the incidence rate in non-carriers family members. Probands were excluded from the analysis to avoid selection bias. Observation time (obs-yrs) was defined as the period from the baseline observation until the first ATE or until the end of the follow-up. Multivariate analysis was performed by Cox proportional hazard regression model to adjust relative risks for clinical relevant covariates including age and sex, hypertension, hyperlipidaemia, diabetes mellitus, body max index (BMI), and active smoking. Results were expressed as hazard ratios with 95% CIs and *P* values. A two-tailed *P* value of less than 0.05 indicated statistical significance. Kaplan–Meier methods were used to describe the ATE risk in the two groups.

## Results

### Subjects

**Fig. 1:**
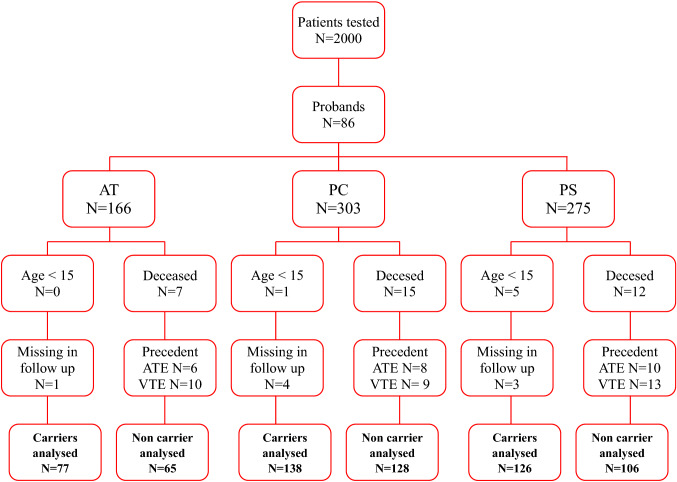
Family cohorths with hereditary antithrombin, protein C, or protein S deficiency

Out of approximately 2000 patients with VTE, 86 unrelated probands were found to be carriers of inherited defects of AT, PC, or PS, and 744 family members were identified as potentially eligible for participation in our investigation. Of these, 104 were excluded because of a prior episode of venous or arterial thrombosis (98), death before enrollment (34), unavailability of information owing to geographic inaccessibility (8), and age younger than 15 years (6). Out of 640 relatives, 142 belonged to families with AT, 266 with PC and 232 with PS defects (Fig. 1); 341 were carriers, and 299 non-carriers of an inherited abnormality. Table 1 details the main demographic characteristics and risk factors for arterial thrombosis of the study population. Age, sex, and smoking habit were equally distributed in carriers and non-carriers of the defects; diabetes mellitus was significantly more prevalent in non-carriers (*P* = 0.04).

**Table 1:** Characteristics of the study population

Variable	Total cohort			Cohort AT			Cohort PC			Cohort PS		
	Deficient	Non-deficient	*P*	Deficient	Non-deficient	*P*	Deficient	Non-deficient	*P*	Deficient	Non-deficient	*P*
Subject N	341	299	Ref	77	65	Ref	138	128	Ref	126	106	Ref
Sex												
F (%)	193 (56.6)	155 (52)	0.23	42 (54.5)	32 (493.2)	1	81 (58.7)	64 (50)	0.18	70 (55.5)	59 (55.6)	1
M (%)	148 (43.4)	144 (48)		35 (45.5)	33 (50.8)		57 (41.3)	64 (50)		56 (44.5)	47 (44.4)	
Median age at enrollment	38	38	0.71	32	42	0.99	40	39	0.58	41	40	0.69
Diabetes mellitus (%)	3 (0.9)	10 (3.3)	0.04	0 (0)	1 (1.5)	0.46	1 (0.72)	2 (1.6)	0.61	2 (1.4)	7 (6.5)	0.08
Hypertension (%)	64 (18.7)	63 (21)	0.70	18 (23.3)	18 (27.2)	0.85	24 (19)	26 (20.4)	0.88	22 (17.5)	19 (17.9)	0.87
Hyperlipidemia (%)	29 (8.5)	17 (5.7)	0.22	11 (13.4)	5 (7.6)	0.30	7 (4.8)	5 (3.9)	0.77	11 (8.8)	7 (6.6)	0.63
Smoking history (%)	78 (22.8)	75 (25)	1	11 (14.3)	21 (31.8)	0.02	35 (25.4)	37 (29.1)	0.68	32 (25.4)	17 (16.1)	0.05
Obesity (%)	124 (36.4)	117 (38)	0.75	28 (36.4)	30 (46.1)	0.32	42 (30.4)	39 (30.7)	1	54 (42.8)	48 (45.3)	0.60
Total ATE	19	7	0.07	1	3	0.32	8	3	0.22	10	1	0.02

A similar proportion (7%) of subjects of either group had used for variable period of time antiplatelet drugs.

### Arterial thromboembolic events

After a follow-up of 4240 patient-years in the carriers group and 3810 in the non-carriers, the mean observation time in each group was 12 years (6–22). ATE occurred in 19 and 7 subjects, respectively, accounting for an annual incidence of 0.45% (95% CI 0.3–0.7) and 0.18% (95%, CI 0.07–0.4) patient-years, respectively; as shown in Table 2, among carriers ATE developed in 1 (1.3%) of the family members with AT deficiency, in 8 (6%) of those with PC defects, and in 10 (8%) of those with PS defects. The corresponding figure in non-carriers was 3 (4.6%), 3 (2.3%), and 1 (0.9%), respectively.

**Table 2:** The median age of the subjects at the time of the first episode of ATE

Variable	Total Cohort			Cohort AT			Cohort PC			Cohort PS		
	Deficient	Non-deficient	*P*	Deficient	Non-deficient	*P*	Deficient	Non-deficient	*P*	Deficient	Non-deficient	*P*
Total ATE	19 (5.6)	7 (2.3)	0.07	1 (1.3)	3 (4.6)	0.32	8 (6.0)	3 (2.3)	0,22	10 (8.0)	1 (0.9)	0.02
MI	4 (1.2)	6 (2.0)	0.56	0 (0)	3 (4.6)	0.09	1 (0.72)	3 (2.3)	0.34	3 (2.4)	0 (0)	0.26
Ischemic Stroke	6 (1.8)	0 (0)	0.03	0 (0)	0 (0)	1	3 (2.2)	0 (0)	0.25	3 (2.4)	0 (0)	0.26
TIA	3 (0.9)	0 (0)	0.26	1 (1.3)	0(0)	1	1 (0.72)	0 (0)	1	1 (0.8)	0 (0)	1
PAD	6 (1.8)	1 (0.3)	0.13	0 (0)	0 (0)	1	3 (2.2)	0 (0)	0.25	3 (2.4)	1 (0.9)	0.63
Median age at event (range)	62 (47–86)	66 (56–79)	0.31	48	66 (56–79)	0.5	66 (60–80)	71 (66–73)	0.29	59 (47–86)	60	1

*MI* myocardial infarction, *TIA* cerebral transient ischemic attack, *PAD* peripheral artery disease

The overall annual incidence of ATE was 0.45% pts-years (95% CI 0.3–0.7) in carriers versus 0.18% pts-years (95% CI 0.07–0.4) in non-carriers. The overall RR of ATE in carriers vs non-carriers was 2.5 (95%CI 1.03–5.8), and it was 0.25 (95% CI 0.03–2.4), 0.25 (95% CI 0.03–2.4), and 2.4 (95% CI 0.6–9) among family members of AT, PC, and PS defects, respectively.

After adjusting for age, sex, hypertension, hyperlipidemia, diabetes, BMI, and smoking history, the HR of ATE in carriers as compared to non-carriers was 4.97 (95% CI 1.5–16.3; *P* = 0.008) (Table 3).

**Table 3:** Multivariate hazard analysis

	*P* =	HR	95% CI
Age in	0.002	1.054	1.019–1.090
Defect carriers	0.008	4.972	1.515–16.320
Sex	0.974	1.017	0.359–2.887
IPA	0.185	2.040	0.712–5.848
Lipid	0.128	2.475	0.770–7.953
Diab	0.009	7.475	1.661–33.647
Obes	0.850	0.907	0.330–2.492
Smoker	0.021	3.575	1.214–10.529

*IPA* hypertension, *Lipid* hyperlipidemia, *Diab* diabetes, *Obes* obesity

**Fig. 2:**
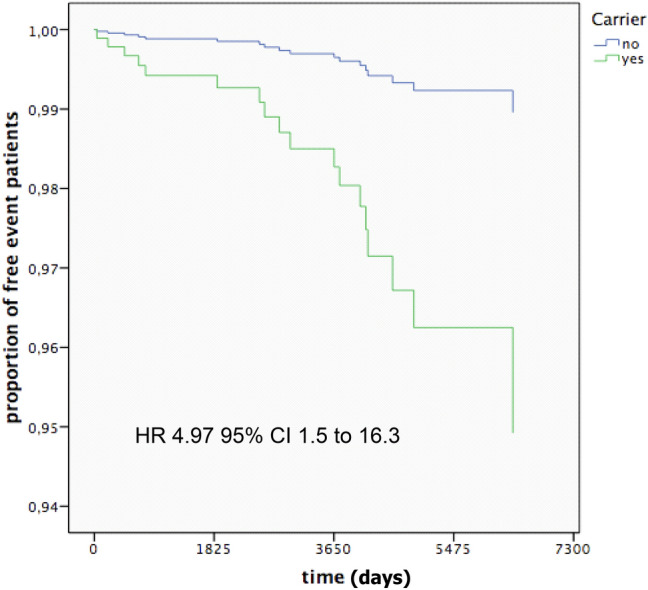
The Kaplan–Meier analysis: event-free survival comparing subjects with and without any deficiency

Figure 2 shows the time-dependent distribution of ATE over time in the two groups.

The risk was higher in individuals younger than 55 years [HR 4.65 (95% CI 1.4–15.0)] compared to the over 55 [HR 1.54 (95% CI 0.6–6.6)].

## Discussion

The results of our prospective cohort study suggest that among family members of probands with natural coagulation inhibitors, carriers of the defect have a remarkably higher risk of ATE than non-carriers. Indeed, after adjusting for confounders, the HR for ATE was almost fivefold higher in carriers than in non-carriers, the risk being higher in younger individuals. These findings are consistent with those obtained in investigations that addressed the risk of subsequent VTE in the same settings [2, 3]. In our recent prospective observation, the incidence of spontaneous thrombosis and that related to risk periods these incidences were 1.2% (0.9–1.7) and 21.2%(13.1–32.4), respectively [13]. These results provide further compelling evidence in support of the association between venous and arterial thrombotic complications [6].

The individuals that were found to exhibit this risk most were the carriers of PC and PS defects. This finding is consistent with those reported by others [11], and may be accounted for by the peculiar mechanisms involved in the PC–PS inhibitory systems [19–22]. Indeed, it is well known that the PC–PS system has not only anticoagulant but also anti-inflammatory and antiapoptotic properties, together with a direct protective effect on the endothelial barrier [19, 20]. Accordingly, PC/PS defects are likely to expose carriers to a higher risk of vascular (both venous and arterial) complications.

Our study results add to the growing evidence that inherited thrombophilia is likely to increase not only the risk of VTE [13], but also of that of atherothrombosis and its complications. They are consistent with those found in the EPCOT study (0.18% in deficient subjects vs 0.03% in the control group) [10] and with those found by Mahmoodi et al. (0.32% in deficient subjects vs 0.19% in non-deficient subjects) in two retrospective family cohort studies on the risk of ATE in carriers of hereditary deficiencies of protein C, protein S, or antithrombin [11].

Not surprisingly, the relative risk was found to diminish among older family members, as the confounding effect of additional risk factors of atherosclerosis increases with age.

The strength of our investigation is the study design. Indeed, we conducted a prospective cohort study in a large number of family members of probands with inherited deficiencies of natural anticoagulants, who were followed up for a long period of time, and all events were documented according to rigorous predefined criteria. Selection bias was prevented by including index patients on the only basis of the occurrence of previous VTE, and excluding family members with a previous episode of arterial thromboembolism. However, because of the relatively low number of absolute events that were recorded in the follow-up of our subjects, the confidence intervals around the 95% CI of the risk ratios were quite large, and thus should induce caution in the interpretation of our results. In addition, as our study was underpowered for the detection of an association between common risk factors for atherosclerosis and ATE, we could not draw any arrive at definite conclusions in this regard. Finally, as we did not enrol subjects belonging to families with other more prevalent abnormalities, such as factor V Leiden and prothrombin mutation, our conclusions cannot apply to carriers of these abnormalities. However, in the previous retrospective study of Mahmoodi et al., no significant interaction was found between concomitant thrombophilic defects (prothrombin G20210A mutation and factor V Leiden) and any deficiency. Future studies on carriers of excess of prothrombotic defects may be useful to evaluate potential risk differences.

In spite of these limitations, our findings confirm the previous reports [11, 12], and suggest that carriers of these abnormalities are at an increased risk not only of venous but also of arterial thrombotic disorders. While providing further evidence in support of the association between venous and arterial thrombosis, our results have the potential to inform the management of carriers of deficiencies of natural anticoagulants: above all by stimulating a lifestyle aimed at preventing the usual cardiovascular risk factors (hypertension, overweight, hypercholesterolemia, etc.) and adopting an early and strict control of the same at onset.
